# Partial Disassembly of the Nuclear Pore Complex Proteins during Semi-Closed Mitosis in *Dictyostelium discoideum*

**DOI:** 10.3390/cells11030407

**Published:** 2022-01-25

**Authors:** Kristina Mitic, Marianne Grafe, Petros Batsios, Irene Meyer

**Affiliations:** Department of Cell Biology, University of Potsdam, Karl-Liebknecht-Str. 24-25, 14476 Potsdam-Golm, Germany; kristina.mitic@uni-potsdam.de (K.M.); marianne.grafe@uni-potsdam.de (M.G.); petros.batsios@googlemail.com (P.B.)

**Keywords:** nuclear pore complex, nucleoporins, semi-closed mitosis, centrosome, *Dictyostelium*

## Abstract

*Dictyostelium* cells undergo a semi-closed mitosis, during which the nuclear envelope (NE) persists; however, free diffusion between the cytoplasm and the nucleus takes place. To permit the formation of the mitotic spindle, the nuclear envelope must be permeabilized in order to allow diffusion of tubulin dimers and spindle assembly factors into the nucleus. In Aspergillus, free diffusion of proteins between the cytoplasm and the nucleus is achieved by a partial disassembly of the nuclear pore complexes (NPCs) prior to spindle assembly. In order to determine whether this is also the case in *Dictyostelium*, we analysed components of the NPC by immunofluorescence microscopy and live cell imaging and studied their behaviour during interphase and mitosis. We observed that the NPCs are absent from the contact area of the nucleoli and that some nucleoporins also localize to the centrosome and the spindle poles. In addition, we could show that, during mitosis, the central FG protein NUP62, two inner ring components and Gle1 depart from the NPCs, while all other tested NUPs remained at the NE. This leads to the conclusion that indeed a partial disassembly of the NPCs takes place, which contributes to permeabilisation of the NE during semi-closed mitosis.

## 1. Introduction

The nucleus of Eukaryotes is surrounded by the nuclear envelope (NE). It consists of two lipid bilayer membranes, the outer one being continuous with the endoplasmic reticulum. The outer and inner nuclear membranes are connected at the embedded nuclear pores, which allow specific transport of cargo between the cytosol and the nucleus. In mitosis, prior to spindle assembly, nuclear envelope break down (NEBD) takes place in most animal cells [1]. This is called an open mitosis. Budding yeast, on the other hand, where the centrosome (known as spindle pole body) is embedded in the NE, performs a fully closed mitosis [2]. The NE stays intact, the spindle is formed inside the nucleus and every component needed for spindle assembly needs to be transported actively into the nucleus. Likewise, there is no nuclear envelope break down in *Dictyostelium*, the nuclear lamina as well as several membrane proteins remain present at the NE throughout mitosis [3]. Nevertheless, after prophase proteins, including tubulin dimers, can diffuse freely into and out of the nucleus [3,4], which is why the terms semi-closed [3] or semi-open [5] are used to describe the situation in the amoeba. In order to assemble a mitotic spindle, the centrosome, which is situated outside of the nucleus in interphase and is tightly attached to the membrane, has to be inserted into the NE. The mechanism behind the insertion process is not known so far; however, previous studies have indicated that the centrosomal core protein CP75 plays an important role in this process [6]. The resulting fenestra in the membrane could be one reason for the leakiness of the NE. A further mechanism has been observed in *Aspergillus nidulans*. Here the nuclear pore complexes (NPCs) are partially disassembled and provide a channel for free diffusion of proteins through the NE [7].

**Figure 1:**
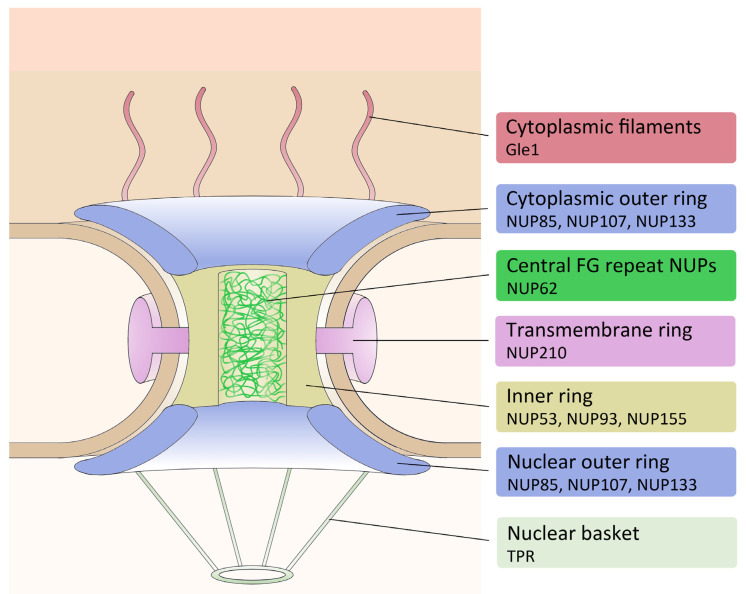
Schematic drawing of the nuclear pore complex. The NPC is organized in several different subcomplexes. The pore consists of an inner ring and two outer rings and is anchored with the membrane via transmembrane nucleoporins. FG-repeat NUPs form a plug inside of the pore to regulate cargo traffic. The nuclear basket projects into the nucleoplasm and eight filaments extend into the cytoplasm. On the right the selected *Dictyostelium* NUPs are mentioned.

The nuclear pore complexes are highly organized protein complexes which are embedded in the nuclear envelope. They show an eightfold symmetry and are composed of several subcomplexes [8] (Figure 1). Scaffold nucleoporins (NUPs) form the central channel (inner ring), flanked by two outer rings, one on the nuclear side, one facing the cytoplasm. Transmembrane proteins anchor the complex with the membrane (transmembrane ring). In the centre, FG-repeat NUPs plug the channel and allow only smaller molecules and proteins to pass freely. Larger proteins need to carry a nuclear localization sequence in order to be actively transported through the pores following a well characterized Ran-dependent process [9]. Eight flexible filaments attached to the cytoplasmic ring (cytoplasmic filaments) and the nuclear basket aid in binding and transporting the cargo [10,11].

In *Dictyostelium* the nuclear pores have been studied extensively using cryo-electron tomography and fantastic snapshots of different stages of the complex during cargo transport have been published [12,13]. However, up until this work little was known about the individual proteins. About twenty NUP-genes have been identified in the genome of *Dictyostelium*. Three of the corresponding proteins have been located at the NE during interphase using GFP-fusion proteins [14]. In addition NUP43 was used as a marker for the NE, but was not characterized in greater detail [15].

In this work we set out to characterize NUPs of *Dictyostelium discoideum* and analyse their dynamic behaviour during the cell cycle. In addition to GFP-fusion proteins we applied a knock-in strategy to study the proteins at an endogenous expression level. The data we gained from both immunofluorescent microscopy and live cell imaging brings us closer to understand the processes occurring during semi-closed mitosis in *Dictyostelium*.

## 2. Materials and Methods

### 2.1. Cloning

In order to express GFP-fusion proteins of NUP53, NUP85, NUP93, NUP133, NUP155, NUP201 and Gle1 in addition to the endogenous proteins, the complete coding regions of the respective NPC proteins were amplified by PCR using linker primers containing SalI (forward primers) and BamHI (reverse primers) restriction sites. The corresponding Dictybase Gene IDs are listed in Table 1. As PCR template AX2 wildtype genomic DNA was used; therefore, introns are included. The resulting products were cloned into plasmid pIS77 [16], which supplies the cells with a G418 resistance. For the C-terminal Neon knock-in constructs of the NPC-proteins NUP53, NUP62, NUP85, NUP93, NUP107 NUP210, TPR and Gle1 the plasmid pIS1272 was used, which was generated from pIS1121 [17] by replacing GFP with a codon-optimized mNeonGreen flanked by a Myc-tag. In addition to Neon-Myc and the Blasticidin S resistance cassette, each knock-in construct contains two sequences for homologous recombination. The first one consisted of the last 500–700 bp of the coding region excluding the stop-codon, which was amplified by PCR using linker primers containing KpnI or SalI (forward primers) and SalI or EcoRI (reverse primers) restriction sites. For the second fragment 500–700 bp of the 3′ region of the genes were amplified by PCR using linker primers containing PstI (forward primers) and BamHI (reverse primers) restriction sites. As template AX2 wildtype genomic DNA was used. The plasmids were linearized by KpnI/BamHI digestion prior to transformation into *Dictyostelium* AX2 wildtype cells.

### 2.2. Cell Culture

For cell culture HL5c medium (Formedium, Hunstanton, UK) was used, supplemented with sterile filtered glucose added after autoclaving. For selection, 10 µg/mL G418 (GFP-overexpressing strains) or 4 µg/mL Blasticidin S (knock-in strains) were added, respectively. For microscopy cells were grown in adherent culture using tissue culture flasks.

### 2.3. Immunofluorescence and Microscopic Imaging

For immunofluorescence microscopy, both GFP and Neon-fusion protein expressing cells were fixed with glutaraldehyde, as described previously [18], and counterstained with the anti-α-tubulin antibody YL1/2 [19] and anti-rat AlexaFluor 568 (Thermo Fisher Scientific, Darmstadt, Germany) or polyclonal antibody against CP224 [18] and anti-rabbit AlexaFluor 568. DNA was stained with DAPI (4,6-diamidino-2-phenylindole). Microscopy and image acquisition was performed at a Zeiss CellObserver HS system or an AxioObserver system equipped with a PlanApo 1.4/100× lens and Axiovision 4.8 software or ZEN Blue Software including the iterative deconvolution module (Carl Zeiss Mikroskopie GmbH, Jena, Germany). In the figures either only one slice of the deconvoluted Z-stack or a brightest point projection of several slices is presented, as mentioned in the description of the individual figures. Fluorescence intensity measurements were done using Fiji. For quantification of the fluorescence signal non-deconvoluted immunofluorescence images of fixed Neon knock-in cells were used. A freehand line was drawn on the mitotic nuclear envelope and the intensity value was measured. For each mitotic cell three measurements were performed, and the mean value was calculated. For normalization the nuclear envelope signal of three interphase cells from the same image was calculated, averaged, and set to 100% as a control. Datasets were tested for normal distribution and *p*-values were calculated using the parametric One-sample *t* test. The significance level of *p* < 0.001 was considered statistically very significant.

### 2.4. Live Cell Imaging

Live cell imaging was performed using a ZEISS Cell Observer SD confocal microscope (Carl Zeiss Microscopy GmbH, Jena, Germany) equipped with the Yokogawa Spinning Disk Unit CSU-X1 and two highly sensitive Evolve EM-CCD cameras (Photometrics, Tucson, AZ, USA). Living cells were imaged with a LCI Plan-Neofluar 63×/1.3 Imm Korr DIC objective and excited with a 488 nm (100 mW) solid state laser using Zeiss AxioVision Rel. 4.9.1 software. Z-stack settings were set to 7–10 slices per stack at a frame spacing of 0.21 µm for NUP62 Neon knock-in and 0.5 µm for NUP210 Neon knock-in cells. Stacks were recorded every 10 s. Maximum intensity projections of image stacks were calculated using Fiji [20]. For live cell imaging cells were settled down in glass bottom dishes (FluoroDish, WPI, Berlin, Germany) for at least 30 min, as described previously [4]. The medium was replaced by Low Fluorescence (LoFlo) medium pH 6.5 (Formedium, Hunstanton, UK) and ascorbic acid was added to a final concentration of 2 mg/mL to reduce phototoxic effects during imaging. Intensity of the NUP signal was measured with Fiji using a summed sliced projection and normalized by the highest value in the time course.

## 3. Results

To study the dynamics of the nucleoporins in *Dictyostelium discoideum*, we chose representative marker components for the individual NPC subcomplexes (Figure 1 and Table 1).

### 3.1. Nuclear Pore Complexes Are Excluded from Nucleoli

**Figure 2:**
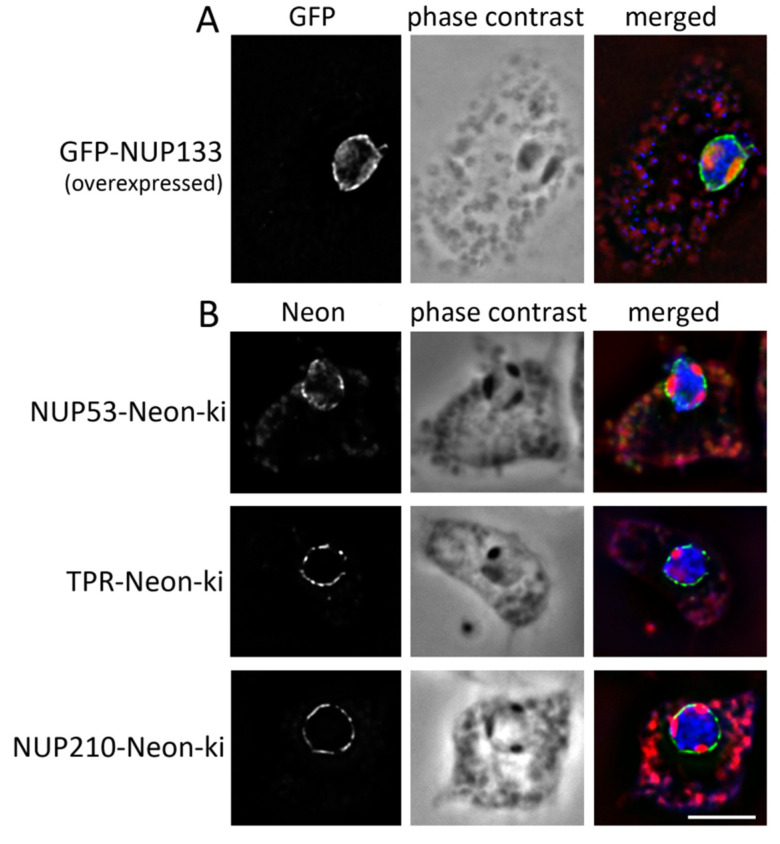
NUPs are absent from the contact sites of the nucleoli with the nuclear envelope. Wide field fluorescence microscopy of glutaraldehyde fixed GFP-NUP133 (**A**) or NUP-Neon-fusion protein (**B**) expressing cells in interphase. Nucleoli are visualized with phase contrast and can be seen as dark areas inside of the nucleus. Overlay (right) of the green fluorescent protein signal (green) with phase contrast (red) and DAPI (blue). Only one Z-slice is shown. Z-stacks are provided as Videos S1–S4. Bar = 5 µm.

We started with the expression of the selected NUPs N-terminally fused to GFP in addition to the endogenous proteins, leading to rather high overexpression. This included Gle1, NUP53, NUP93, NUP85, NUP133, NUP155 and NUP210. All GFP-fusion proteins localized to the nuclear envelope. In addition, transmembrane nucleoporin NUP210 could be found at the endoplasmic reticulum (data not shown). Overexpressed N-terminally tagged GFP-NUP133 was not only observed at the nuclear envelope but, in addition, inside the nucleoli (Figure 2A). We cannot exclude that this is an overexpression effect. Therefore, we wanted to analyse NUP localisation with fusion proteins at endogenous expression levels using the brighter green fluorescing mNeonGreen-Myc tag (short: Neon).

Unfortunately, we were not able to generate a NUP133-Neon knock-in or an NUP155-Neon strain. In our knock-in strategy, the tag must be inserted after the coding sequence of the protein of interest, in order to express the fusion protein under the endogenous promoter. The failure of our knock-in attempts could be explained by C-terminally tagged NUP133 or NUP155 proteins not being fully functional.

For all other tested NPC-components, our Neon-fusion knock-in strategy worked nicely. We therefore analysed fusion proteins of altogether eight NPC-components (NUP53, NUP62, NUP85, NUP93, NUP107, NUP210, Gle1, TPR) with a C-terminal Neon-tag under control of the respective endogenous promoter. We found that, in all these strains, the Neon signal was not equally distributed at the NE. When we compared the GFP-signal with the localization of the nucleoli, which are visible as dark areas inside the nucleus in phase contrast microscopy, we observed that most fusion proteins were absent from the contact sites of the nucleoli with the nuclear envelope (Figure 2B and Figure S1).

### 3.2. Some NUPs Localize to the Centrosome

**Figure 3:**
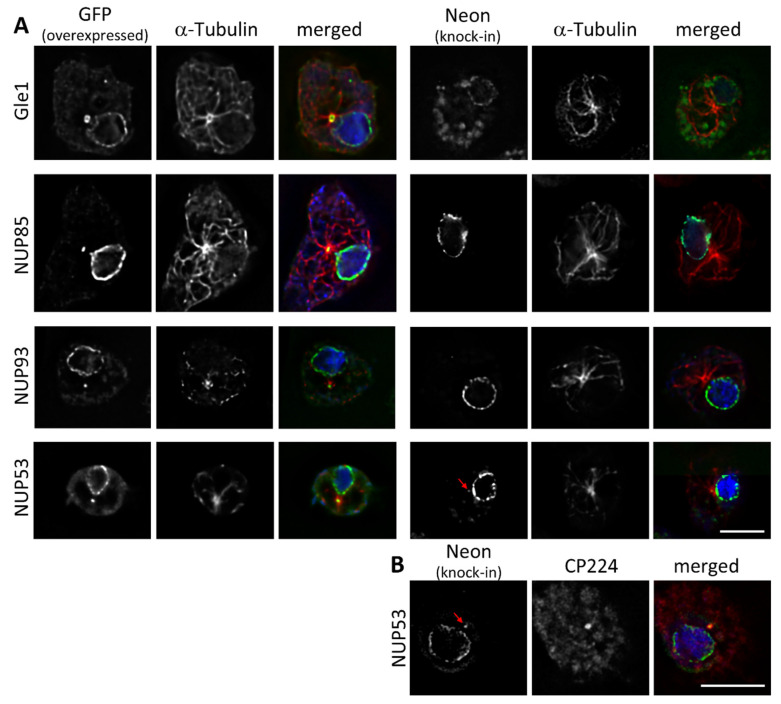
Centrosomal localization of overexpressed Gle1, NUP53, NUP85 and NUP93. At endogenous expression levels only NUP53-Neon can be found at the centrosome (red arrow). Immunofluorescence microscopy of GFP-NUP (overexpressed, left side) or NUP-Neon (knock-in, right side) expressing cells in interphase fixed with glutaraldehyde, stained with either anti-α-Tubulin and secondary antibody anti-rat-AlexaFluor-568 (**A**) or anti-CP224 and secondary antibody anti-rabbit-AlexaFluor-568 (**B**). Only the one focus plane containing the centrosome is presented. Overlay of the Green Fluorescent Protein signal (green) with α-Tubulin or CP224 (red) and DAPI (blue). Bar = 5 µm.

Some of the overexpressed GFP-fusion proteins show signals that colocalize with the microtubule organizing centre (MTOC), which becomes visible after staining with tubulin antibodies (Figure 3A). In *Dictyostelium* the centrosome, which is the only MTOC during interphase, consists of a layered core structure surrounded be the corona and is closely attached to the nucleus [21]. While GFP-NUP53 localized to the centrosome of every cell, the GFP-fusion proteins of Gle1, NUP93 and NUP85 showed this localization only in cells with brighter GFP fluorescence, indicating higher expression levels. Only in very few cells GFP-NUP133 and GFP-NUP155 could also be observed at the centrosome (Figure S2).

GFP-NUP53, GFP-NUP85 and GFP-NUP93 showed rather small dot-like signals at the centrosome, indicating a localisation at its core structure. Gle1 on the other hand is more likely associated to the corona, as larger and doughnut-shaped GFP-signals could be seen.

When observing the fusion proteins at endogenous protein expression levels, only NUP53-Neon showed a weak, but in every cell visible centrosomal signal (Figure 3A). When staining the cells with an antibody against the centrosomal corona protein CP224, NUP53- Neon is visible as a small spot in the centre of the larger, doughnut shaped signal of CP224 indicating that NUP53 is part of the centrosomal core structure (Figure 3B). All other C-terminally tagged Neon-fusion proteins were absent from interphase centrosomes.

### 3.3. NUPs Show Divergent Behaviour during Mitosis

**Figure 4:**
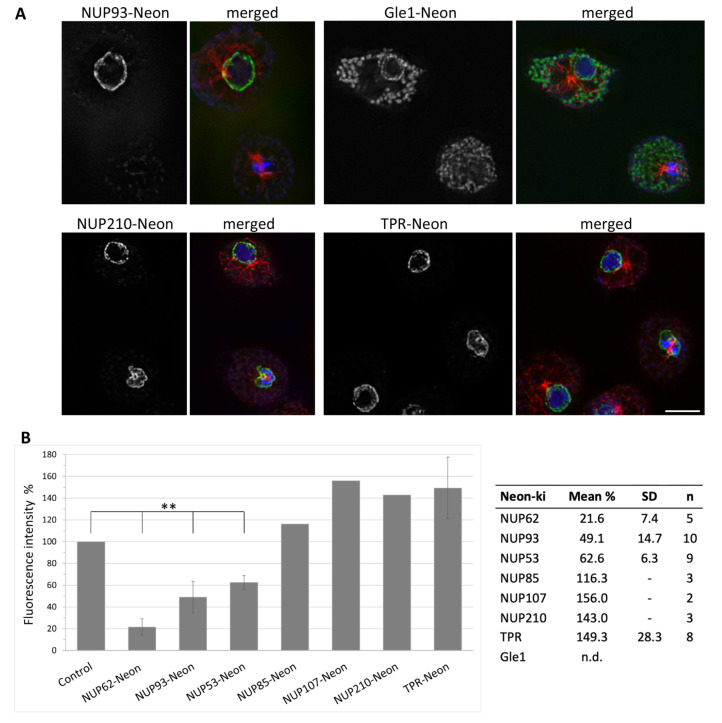
NUP93 and Gle1 disappear from the nuclear envelope during mitosis, whereas NUP210 and TPR remain. (**A**) Immunofluorescence microscopy of Neon knock-in cells fixed with glutaraldehyde, stained with anti-α-Tubulin and secondary antibodies anti-rat-AlexaFluor-568. One metaphase cell and neighbouring interphase cells are shown. Maximum intensity projection of the slices containing the nuclei. Overlay of the green fluorescent protein signal (green) with α-Tubulin (red) and DAPI (blue). Bar = 5 µm. (**B**) Quantification of the fluorescence intensity signal of respective NUP-Neon knock-in cells during metaphase. Columns represent the mean fluorescence intensity measured from widefield microscopy images. For each mitotic cell the fluorescence intensity of a neighboring interphase cell was measured and normalized to 100% (Control). Mean values and standard deviation (SD) are shown in the table. Due to a lack of Gle1 signal during metaphase, there are no measurements available. *n* = sample number, SD was calculated only for *n* > 4; n.d. = not defined. Error bars represent standard deviation of the mean. One-sample *t* test; ** *p* value < 0.001.

In *Aspergillus*, a partial disassembly of nuclear pore complexes during mitosis has been described [7]. Therefore, we also studied the mitotic behaviour of the NPC proteins in *Dictyostelium*. In the Neon knock-in strains of Gle1 and NUP93 (Figure 4A), as well as NUP62 and NUP53 (Figure S3), we observed a strong reduction of the Neon signal around the nucleus in metaphase. For the remaining components, however, a consistent fluorescent signal could be observed at the nuclear envelope throughout mitosis, indicating that at least parts of the pore complex stay assembled. This behaviour could be observed for NUP210, NUP85, NUP133, NUP155 and the nuclear basket protein TPR (Figure 4 and Figure S3).

To quantify the decrease of the proteins at the NE in the knock-in strains, we measured the mean intensity of the Neon signal at the NE of metaphase cells in comparison to the signal of interphase cells (Figure 4B). In Gle1-Neon metaphase cells, the NE was not visible at all, therefore we were not able to collect the data. The strongest reduction that could be measured was for the central FG protein NUP62. Here, the signal was, on average, reduced to 22% of its interphase intensity. The signal of NUP53-Neon and NUP93, two components of the inner ring complex, were also clearly reduced. For these, average mean intensity went down to 63% or 48%, respectively. By contrast, an increased Neon signal could be observed for the knock-in strains of NUP85, NUP107, NUP210 and TPR, probably due to a reduced diameter of the nuclei leading to a higher density of the NPCs.

**Figure 5:**
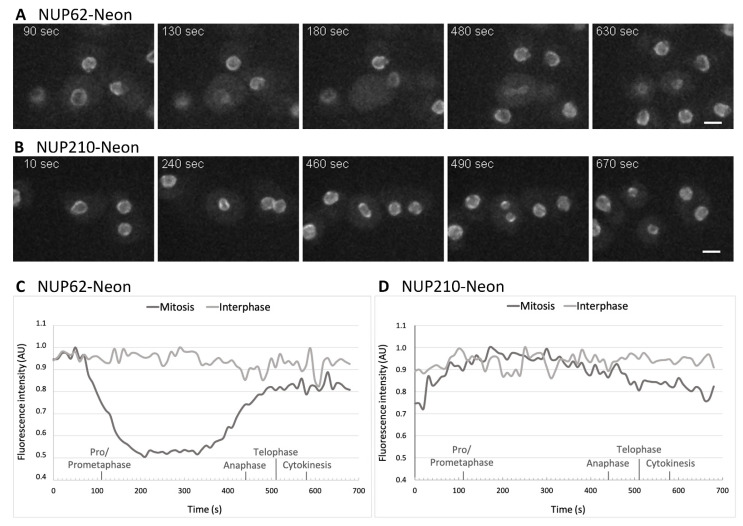
Dynamic changes of the NPCs during mitosis. Live cell spinning disk confocal microscopy of NUP62 Neon knock-in cells (**A**,**C**) and NUP210 Neon knock-in cells (**B**,**D**) with excitation at 488 nm. Selected time points of Videos S5 and S6 are shown. (**A**) The central FG NUP62 disappears from mitotic NPCs at the onset of mitosis and reappears right before karyokinesis at time point 10:50 min. (**B**) The transmembrane protein NUP210 remains at the NPC during the entire cell cycle. Maximum intensity projections of 7–10 slices per image stack (z-distance 0.21 µm (**A**) and 0.5 µm (**B**), respectively). Stacks were recorded every 10 s. Bar = 5 µm. (**C**,**D**): Intensity of the nuclear Neon signal of the of mitotic and interphase cells shown in (**A**,**B**). Estimated start points of mitotic phases are indicated on the time line (x-axis) based on cell morphology and nuclear shape observed in the fluorescence channel (specific and background signal). Intensity of the NUP signal was measured with Fiji using a summed sliced projection and normalized to the maximal value.

For live cell imaging we chose knock-in strains of the transmembrane protein NUP210 and the central FG-repeat protein NUP62. While the NUP210-Neon signal persists at the nuclear envelope throughout all mitotic stages (Figure 5B,D, Video S6), NUP62-Neon leaves its localization around the nucleus at the onset of mitosis and more of the fluorescent protein can be observed in the cytoplasm (Figure 5A,C, Video S5). In anaphase NUP62-Neon starts to reappear at the nuclear envelope regaining normal intensity after the separation of the two daughter nuclei in early telophase. 

### 3.4. During Mitosis Some NUPs Concentrate at Spindle Poles

**Figure 6:**
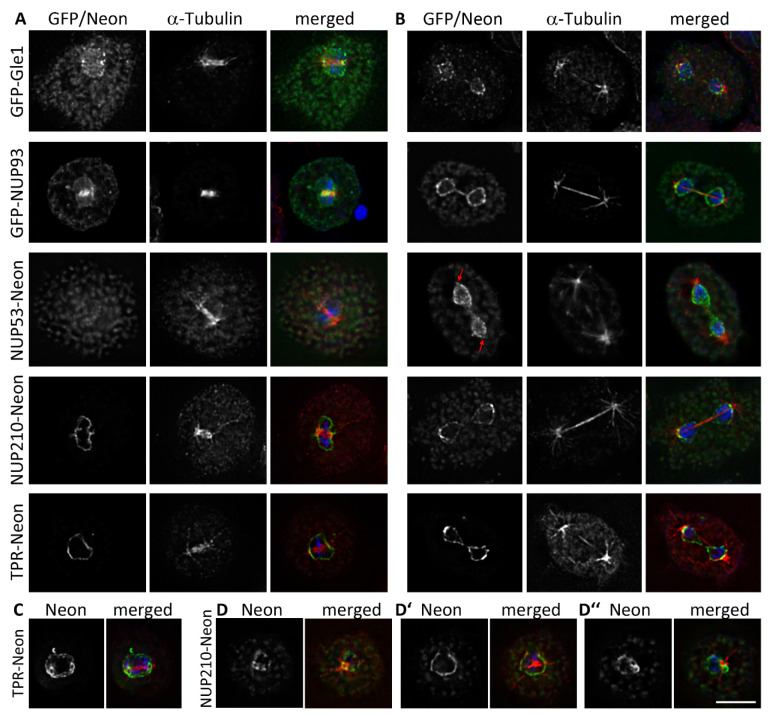
Overexpressed Gle1 and NUP93 localize to spindle poles. NUP53 Neon is absent from spindle poles in metaphase but reappears in telophase (red arrows). Neon-fusion proteins of NUP210 and TPR are concentrated close to the spindle poles and form a ring around the poles in metaphase. Immunofluorescence microscopy of GFP-NUP or NUP-Neon-fusion protein expressing cells in interphase fixed with glutaraldehyde, stained with anti-α-Tubulin and secondary antibodies anti-rat-AlexaFluor-568. In (**A**) only the slice with focus on the metaphase spindle is shown. In (**B**,**C**) projections of several Z-slices are presented. (**D**) shows three different slices of the same NUP210-Neon metaphase cell with a tilted spindle (**D**: focus on the left pole, **D’** focus on the centre, **D’’**: focus on the right pole). Overlay of the green fluorescent protein signal (green) with α-Tubulin (red) and DAPI (blue). Bar = 5 µm.

When overexpressed, the GFP-fusion proteins of Gle1 and NUP93 could be found at the spindle poles in metaphase (Figure 6A) and telophase (Figure 6B); GFP-NUP93 in addition also at the kinetochores of metaphase spindles. As in interphase cells, the centrosomal localization cannot be observed for Neon-fusion proteins expressed at endogenous levels (Figure 4 and Figure S4). NUP53-Neon, which is present at the centrosomes in interphase, disappears from the mitotic centrosome in an early stage of mitois and is absent from the spindle poles during metaphase (Figure 6A). In telophase the centrosomal signal has reappeared together with the NE staining (Figure 6B). TPR-Neon and NUP210-Neon showed increased fluorescence around the poles leading to a ring-like appearance if the metaphase spindle is not in a horizontal position but slightly tilted (Figure 6C,D). In later stages TPR-Neon and also NUP210-Neon could be found concentrated close to the spindle poles, which, at this mitotic stage, are already positioned back outside of the nuclear envelope (Figure 6B).

## 4. Discussion

In order to increase our knowledge of the nuclear envelope in *Dictyostelium*, we selected components of every NPC subcomplex and expressed them as fluorescent fusion proteins. Our first observation was that in interphase cells all studied NUPs were distributed unevenly but were excluded from the area where the nucleoli are attached, indicating that there are no pore complexes in these regions. In the nucleoli the ribosomal subunits are assembled. Therefore, these large complexes must be transported out of the nucleoli into the nuclear matrix prior to their export through the nuclear pore complexes. In animal cells there is mostly one nucleolus, which is located centrally in the nucleus and has no contact to the nuclear envelope. Thus, there is no connection between the nuclear envelope and the nucleolus. In budding yeast, on the other hand, the nucleolus occupies about a third of the nuclear volume and is located at the periphery of the nucleus. Most NUPs are present in this area; only the basket proteins Mlp1 and Mlp2 are excluded from nucleoli-associated pore complexes [22,23,24]. This indicates that there are different types of NPCs present in yeast and the NPCs in the vicinity of the nucleoli are devoid of the nuclear basket structure. In *Dictyostelium,* however, all analysed NUPs, which included at least one component of every subcomplex, showed the same distribution when expressed at endogenous levels, all of them being absent from the nucleolar attachment sites. We can only speculate about the mechanism behind this exclusion of NPCs from this area. It was previously observed that, in *Dictyostelium,* the MAN1-like transmembrane protein Src1 is concentrated at the nuclear envelope close to the nucleoli, suggesting a nucleolar function [25]. This makes Src1 a good candidate to play a role in the exclusion of NPCs from the nucleolar attachment sites. In a search for interaction partners using the BioID method, lamin protein NE81 was identified. This connection to the lamin network could provide the mechanical stability for Src1 to organize the nucleoli and exclude the pore complexes from this NE-region, potentially by condensing the lamin network. Other likely interactors for Src1 identified by BioID are NUP93, NUP107 and NUP133 (Batsios, unpublished data), which provides a possible explanation of why NUP133 can be found inside the nucleoli when overexpressed.

In mitotic *Dictyostelium* cells the NUPs showed two different behaviours with some of them staying at the NE and some disappearing. This indicates that a partial disintegration of the pore complexes in mitosis takes place in *Dictyostelium*, which then allows free diffusion of proteins through the remaining parts of the pores complexes during semi-closed mitosis. In open mitosis disassembly of the NPCs is also a key event during NEBD. Here, the PLK1 mediated phosphorylation of NUP98 and subsequent dissociation from the NE correlates with the leakage of nuclear proteins into the cytoplasm in prophase [26,27]. Phosphorylation of NUP53 then triggers disassembly of the central NPC scaffold, breaking the barrier of the NE. While, in semi-closed mitosis, the disintegration process apparently stops at this stage, the subcomplexes of the outer rings leave the NE in open mitosis as well.

In *Dictyostelium* the disassembly of the NPCs is quite similar to the corresponding process *Aspergillus* [7]. In both species, the analysed central FG NUPs are released from the pore complex, which is prerequisite to open the pores and enable free diffusion of proteins that are crucial for constructing the mitotic spindle inside the nucleus. In addition, some components of the inner ring complex also disassemble. In *Dictyostelium* this includes NUP93 and NUP53 (for the latter no orthologue is known in *Aspergillus*). However, not all *Dictyostelium* NPC proteins correspond in their behaviour to *Aspergillus*. In the fungus the cytoplasmic filament protein Gle1 is a permanent component of the nuclear pores, while it is dispersed during mitosis in *Dictyostelium*.

Quite surprising is the mitotic presence of the basket protein TPR at the nuclear envelope in *Dictyostelium*. In animal cells, the basket protein is among the first proteins to disappear with the NEBD and is reassembled only in G1 [28]. In *Aspergillus* the partial disassembly during semi-closed mitosis includes the basket proteins Nup2, Mlp2 and the TPR orthologue Mlp1. While Mlp1 and Mpl2 diffuse into the cytoplasm, Nup2 stays within the nucleus by binding to chromatin [7]. A similar scenario is possible for TPR in *Dictyostelium*. In mammalian cells, it has been shown that TPR connects the pore complexes with the nuclear lamina by interacting with lamin B1 [29]. If this is also the case for TPR in *Dictyostelium*, it is possible that the protein disassembles from NPC after all but stays at the nuclear envelope only through its binding to the *Dictyostelium* lamin protein NE81.

Interestingly TPR, and after metaphase also the transmembrane protein NUP210 are enriched in the area close to the spindle poles. Not all NUPs are concentrated there, hence the two proteins are not only part of the nuclear pore complexes but gain this additional localization through interaction with non-NUP proteins. A likely candidate for the latter is the nuclear membrane protein Sun1, which is also enriched around the mitotic centrosome and is known to play a role in anchoring the centrosome to the NE in interphase [15,30]. This leads to the idea that TPR and/or NUP210 together with Sun1 could play a role in tethering the mitotic centrosome within the nuclear envelope. In *S. cerevisiae* the transmembrane NUP Ndc1 locates to both the NPC and the SPB [31]. The *S. pombe* Ndc1 orthologue Cut11 locates to the NPC and transiently to the SPB during mitosis [32]. It has also been shown to interact with Sun1 orthologue Sad1. So far, no Ndc1 orthologue could be identified in *Dictyostelium* and with NUP210 being the only known transmembrane NUP in the amoeba, it is possible that NUP210 adopts the function of the yeast proteins Ndc1/Cut11 in connecting the centrosome with the nuclear membrane. In addition, NUP210 starts accumulating close to the centrosome in anaphase, so it could also be involved in the centrosome’s movement back into the cytoplasm and possibly also in resealing the nuclear membrane after completion of chromosome segregation.

Apart from their role as components of the nuclear pore complexes and, therefore, in nuclear import and export, additional functions have been identified for several NUPs. NUP62 for example is associated with spindle microtubules. It plays a role in the spindle assembly checkpoint and is, therefore, important for the maintenance of chromosome integrity [33,34]. The complex Nup107/Nup160 also has a connection to the microtubule cytoskeleton. Those NUPs have been shown to promote spindle assembly through Ran-GTP-regulated nucleation of microtubules by γ-TuRC at kinetochores [35]. In *Xenopus tropicalis* both Nup93 and Nup188 are localized at centrosomes and basal bodies and are required for ciliary function [36]. Finally, Gle1 has also been found at the centrosome and basal bodies of human cells, with its down regulation leading to defects in centrosome integrity and ciliary motility [37].

In *Dictyostelium,* Gle1 localizes to the centrosome in interphase, as well as to the spindle poles, during mitosis, but only when overexpressed. It is possible that if expressed at endogenous levels, only few single molecules reside at the centrosome and that this leads to a signal too weak to be observed by our methods. The same holds true for NUP85 and NUP93 but compared to Gle1 they show a more central localization at the centrosome, probably at its core structure when expressed at higher levels. Even though this localisation might be artificial due to the overexpression, it could potentially tell us something about interaction partners that anchor them at the centrosome. A good candidate for this would be NUP53, since this inner ring NUP was the only protein in this study which could be found at the centrosome of interphase cells when expressed at endogenous protein levels. In addition, NUP93 and NUP53 are part of the same subcomplex and are known to interact in animal cells [38,39]. Thus, it is not far-fetched to presume that NUP93 could also bind to the centrosomal fraction of NUP53 in *Dictyostelium*. Due to its central localization it can be assumed that NUP53 interacts with one of the core components, either with Cep192 [40] in the outer layers, or one of the central layer proteins CP39, CP75 or CP91 [16]. For CP75 it has been speculated to play a role in the insertion of the centrosome into the nuclear envelope at the onset of mitosis, and it is conceivable that NUP53 assists the centrosomal protein in this function by virtue of its membrane-shaping properties [41]. It will be exciting to analyse this in future studies and, hopefully, to identify the reason why this NUP resides at the centrosome. In addition, more work needs to be done to correlate the processes of centrosome insertion and partially disassembly of the NPCs with the leakiness of the NE to clarify the chronology of these events in semi-closed mitosis.

## 5. Conclusions

In this work, we analysed individual components of *Dictyostelium* nuclear pore complexes. We learned that the NPCs are excluded from the regions where the nucleoli are attached to the NE, but the mechanism behind this has yet to be elucidated. We found that several NUPs have a connection to the centrosome in interphase and the spindle poles during mitosis. They could play a role in the process of centrosome insertion into the NE prior to spindle assembly and the tethering of spindle poles in the membrane during chromosome separation. In addition, we observed that in the beginning of mitosis the NPCs are partially disassembled, a mechanism so far only described for fungi, indicating that it was not an invention of the Opisthokonta. This, presumably together with the fenestra around the inserted centrosome, facilitates free diffusion of proteins between the cytoplasm and the nucleus in semi-closed mitosis in *Dictyostelium*.

## Figures and Tables

**Table 1:** Selected NUPs, their Dictybase Gene ID and the corresponding NPC complex.

NPC Complex	Protein	Dictybase ID
Cytoplasmic filaments	Gle1	DDB_G0267918
	NUP53	DDB_G0276109
Inner ring	NUP93	DDB_G0267480
	NUP155	DDB_G0291163
	NUP85	DDB_G0285415
Outer ring	NUP107	DDB_G0285579
	NUP133	DDB_G0287497
Central FG	NUP62	DDB_G0274587
Nuclear basket	TPR	DDB_G0288073
Transmembrane	NUP210	DDB_G0288545

## Data Availability

Genomic data on the genes is available at https://dictybase.org accessed on 31 December 2021. Further original data are available upon request to the corresponding author.

## Supplementary Materials

The following supporting information can be downloaded at: https://www.mdpi.com/article/10.3390/cells11030407/s1, Figure S1: NUPs are absent from the contact sites of the nucleoli with the nuclear envelope. Wide field fluorescence microscopy of glutaraldehyde fixed GFP-NUP or NUP-Neon-fusion protein expressing cells in interphase. Only one Z-slice is shown. Nucleoli are visualized with phase contrast and can be seen as dark areas inside of the nucleus. Overlay (right) of the green fluorescent protein signal (green) with phase contrast (red) and DAPI (blue). Bar = 5 µm. Figure S2: When overexpressed GFP-NUP133 and GFP-NUP155 localize to the centrosome in some, but not all cells. In knock-in strains expressing C-terminally tagged NUP62, NUP107, NUP210 and TPR don’t show centrosomal localization. Immunofluorescence microscopy of in interphase fixed with glutaraldehyde, stained with anti-α-Tubulin and secondary antibody anti-rat-AlexaFluor-568. Only the one focus plane holding the centrosome is presented. Overlay of the green fluorescent protein signal (green) with α-Tubulin (red) and DAPI (blue). Bar = 5 µm. Figure S3: While NUP53-Neon and NUP62-Neon are absent from the NE in metaphase cells, NUP85-Neon, NUP107-Neon, GFP-NUP133 and GFP-NUP155 are present throughout mitosis. Immunofluorescence microscopy of Neon knock-in cells or GFP overexpressing cells with glutaraldehyde, stained with anti-α-Tubulin and secondary antibodies anti-rat-AlexaFluor-568. One metaphase cell and a neighbouring interphase cell are shown. Maximum intensity projection of the slices containing the nucleus. For GFP-NUP133 and GFP-NUP155 a montage of two cells taken from the same image is shown. Overlay of the green fluorescent protein signal (green) with α-Tubulin (red) and DAPI (blue). Bar = 5 µm. Figure S4: When expressed at endogenous levels NUP93 and Gle1 are not enridged at telophase spindle poles. Immunofluorescence microscopy of Neon knock-in cells with glutaraldehyde, stained with anti-α-Tubulin and secondary antibodies anti-rat-AlexaFluor-568. One telophase cell and a neighbouring interphase cell are shown. Maximum intensity projection of the slices containing the nucleus. Overlay of the green fluorescent protein signal (green) with α-Tubulin (red) and DAPI (blue). Bar = 5 µm. Video S1: Z-stack of the NUP133-Neon cell shown in Figure 2A. Left: NUP133-Neon; middle: Overlay of NUP133 (green), microtubules (red), DAPI (blue) and phase contrast; right: phase contrast. Video S2: Z-stack of the NUP53-Neon cell shown in Figure 2B. Left: NUP53-Neon; middle: Overlay of NUP53 (green), microtubules (red), DAPI (blue) and phase contrast; right: phase contrast. Video S3: Z-stack of the NUP210-Neon cell shown in Figure 2B. Left: NUP210-Neon; middle: Overlay of NUP210 (green), microtubules (red), DAPI (blue) and phase contrast; right: phase contrast. Video S4: Z-stack of the TPR-Neon cell shown in Figure 2B. Left: TPR-Neon; middle: Overlay of TPR (green), microtubules (red), DAPI (blue) and phase contrast; right: phase contrast. Video S5: Live cell imaging of NUP62 Neon knock-in expressing *Dictyostelium* cells during mitosis (see Figure 5). The central FG NPC protein NUP62 starts dissociating from the NPCs at the onset of mitosis at time point 90 sec. It firstly reassembles at the nuclear envelope in anaphase at 440 sec and is reappeared after karyokinesis in telophase.. Z-stacks of 7 slices with slice spacing of 0.21 µm were recorded every 10 s. Maximum intensity Z-projections of image stacks were calculated in Fiji. Video S6: Live cell imaging of NUP210 Neon knock-in expressing *Dictyostelium* cells during mitosis (see Figure 5). The transmembrane NPC protein NUP210 is localizing to the NPCs during the whole cell cycle. Thus, it is permanently located at the nuclear envelope. Z-stacks of 10 slices with slice spacing of 0.5 µm were recorded every 10 s. Maximum intensity Z-projections of image stacks were calculated in Fiji.

## References

[1] Güttinger S., Laurell E., Kutay U. (2009). Orchestrating Nuclear Envelope Disassembly and Reassembly during Mitosis. Nat. Rev. Mol. Cell Biol..

[2] Kubai D.F. (1975). The Evolution of the Mitotic Spindle. Int. Rev. Cytol..

[3] Batsios P., Gräf R., Koonce M.P., Larochelle D.A., Meyer I. (2019). Nuclear Envelope Organization in *Dictyostelium discoideum*. Int. J. Dev. Biol..

[4] Samereier M., Meyer I., Koonce M.P., Gräf R. (2010). Live Cell-Imaging Techniques for Analyses of Microtubules in *Dictyostelium*. Methods Cell Biol..

[5] O’Day D.H., Budniak A. (2015). Nucleocytoplasmic Protein Translocation during Mitosis in the Social Amoebozoan *Dictyostelium discoideum*. Biol. Rev. Camb. Philos. Soc..

[6] Meyer I., Peter T., Batsios P., Kuhnert O., Krüger-Genge A., Camurça C., Gräf R. (2017). CP39, CP75 and CP91 Are Major Structural Components of the *Dictyostelium* Centrosome’s Core Structure. Eur. J. Cell Biol..

[7] Osmani A.H., Davies J., Liu H.-L., Nile A., Osmani S.A. (2006). Systematic Deletion and Mitotic Localization of the Nuclear Pore Complex Proteins of Aspergillus Nidulans. Mol. Biol. Cell.

[8] Frenkiel-Krispin D., Maco B., Aebi U., Medalia O. (2010). Structural Analysis of a Metazoan Nuclear Pore Complex Reveals a Fused Concentric Ring Architecture. J. Mol. Biol..

[9] Lange A., Mills R.E., Lange C.J., Stewart M., Devine S.E., Corbett A.H. (2007). Classical Nuclear Localization Signals: Definition, Function, and Interaction with Importin Alpha. J. Biol. Chem..

[10] Franke W.W., Scheer U., Krohne G., Jarasch E.D. (1981). The Nuclear Envelope and the Architecture of the Nuclear Periphery. J. Cell Biol..

[11] Panté N., Aebi U. (1996). Sequential Binding of Import Ligands to Distinct Nucleopore Regions during Their Nuclear Import. Science.

[12] Beck M., Förster F., Ecke M., Plitzko J.M., Melchior F., Gerisch G., Baumeister W., Medalia O. (2004). Nuclear Pore Complex Structure and Dynamics Revealed by Cryoelectron Tomography. Science.

[13] Beck M., Lucić V., Förster F., Baumeister W., Medalia O. (2007). Snapshots of Nuclear Pore Complexes in Action Captured by Cryo-Electron Tomography. Nature.

[14] Beck M., Medalia O. (2008). Structural and Functional Insights into Nucleocytoplasmic Transport. Histol. Histopathol..

[15] Xiong H., Rivero F., Euteneuer U., Mondal S., Mana-Capelli S., Larochelle D., Vogel A., Gassen B., Noegel A.A. (2008). *Dictyostelium* Sun-1 Connects the Centrosome to Chromatin and Ensures Genome Stability. Traffic Cph. Den..

[16] Schulz I., Erle A., Gräf R., Krüger A., Lohmeier H., Putzler S., Samereier M., Weidenthaler S. (2009). Identification and Cell Cycle-Dependent Localization of Nine Novel, Genuine Centrosomal Components in *Dictyostelium discoideum*. Cell Motil. Cytoskelet..

[17] Pitzen V., Askarzada S., Gräf R., Meyer I. (2018). CDK5RAP2 Is an Essential Scaffolding Protein of the Corona of the *Dictyostelium* Centrosome. Cells.

[18] Gräf R., Euteneuer U., Ho T.-H., Rehberg M. (2003). Regulated Expression of the Centrosomal Protein DdCP224 Affects Microtubule Dynamics and Reveals Mechanisms for the Control of Supernumerary Centrosome Number. Mol. Biol. Cell.

[19] Wehland J., Willingham M.C., Sandoval I.V. (1983). A Rat Monoclonal Antibody Reacting Specifically with the Tyrosylated Form of Alpha-Tubulin. I. Biochemical Characterization, Effects on Microtubule Polymerization in vitro, and Microtubule Polymerization and Organization in Vivo. J. Cell Biol..

[20] Schindelin J., Arganda-Carreras I., Frise E., Kaynig V., Longair M., Pietzsch T., Preibisch S., Rueden C., Saalfeld S., Schmid B. (2012). Fiji: An Open-Source Platform for Biological-Image Analysis. Nat. Methods.

[21] Gräf R., Grafe M., Meyer I., Mitic K., Pitzen V. (2021). The *Dictyostelium* Centrosome. Cells.

[22] Miné-Hattab J., Taddei A. (2019). Physical Principles and Functional Consequences of Nuclear Compartmentalization in Budding Yeast. Curr. Opin. Cell Biol..

[23] Galy V., Gadal O., Fromont-Racine M., Romano A., Jacquier A., Nehrbass U. (2004). Nuclear Retention of Unspliced MRNAs in Yeast Is Mediated by Perinuclear Mlp1. Cell.

[24] Niepel M., Molloy K.R., Williams R., Farr J.C., Meinema A.C., Vecchietti N., Cristea I.M., Chait B.T., Rout M.P., Strambio-De-Castillia C. (2013). The Nuclear Basket Proteins Mlp1p and Mlp2p Are Part of a Dynamic Interactome Including Esc1p and the Proteasome. Mol. Biol. Cell.

[25] Batsios P., Ren X., Baumann O., Larochelle D.A., Gräf R. (2016). Src1 Is a Protein of the Inner Nuclear Membrane Interacting with the *Dictyostelium* Lamin NE81. Cells.

[26] Linder M.I., Köhler M., Boersema P., Weberruss M., Wandke C., Marino J., Ashiono C., Picotti P., Antonin W., Kutay U. (2017). Mitotic Disassembly of Nuclear Pore Complexes Involves CDK1- and PLK1-Mediated Phosphorylation of Key Interconnecting Nucleoporins. Dev. Cell.

[27] Kutay U., Jühlen R., Antonin W. (2021). Mitotic Disassembly and Reassembly of Nuclear Pore Complexes. Trends Cell Biol..

[28] Hase M.E., Cordes V.C. (2003). Direct Interaction with Nup153 Mediates Binding of Tpr to the Periphery of the Nuclear Pore Complex. Mol. Biol. Cell.

[29] Fišerová J., Maninová M., Sieger T., Uhlířová J., Šebestová L., Efenberková M., Čapek M., Fišer K., Hozák P. (2019). Nuclear Pore Protein TPR Associates with Lamin B1 and Affects Nuclear Lamina Organization and Nuclear Pore Distribution. Cell. Mol. Life Sci. CMLS.

[30] Schulz I., Baumann O., Samereier M., Zoglmeier C., Gräf R. (2009). *Dictyostelium* Sun1 Is a Dynamic Membrane Protein of Both Nuclear Membranes and Required for Centrosomal Association with Clustered Centromeres. Eur. J. Cell Biol..

[31] Lau C.K., Giddings T.H., Winey M. (2004). A Novel Allele of Saccharomyces Cerevisiae NDC1 Reveals a Potential Role for the Spindle Pole Body Component Ndc1p in Nuclear Pore Assembly. Eukaryot. Cell.

[32] West R.R., Vaisberg E.V., Ding R., Nurse P., McIntosh J.R. (1998). Cut11(+): A Gene Required for Cell Cycle-Dependent Spindle Pole Body Anchoring in the Nuclear Envelope and Bipolar Spindle Formation in Schizosaccharomyces Pombe. Mol. Biol. Cell.

[33] Wu Z., Jin Z., Zhang X., Shen N., Wang J., Zhao Y., Mei L. (2016). Nup62, Associated with Spindle Microtubule Rather than Spindle Matrix, Is Involved in Chromosome Alignment and Spindle Assembly during Mitosis. Cell Biol. Int..

[34] Chien M.-L., Lai J.-H., Lin T.-F., Yang W.-S., Juang Y.-L. (2020). NUP62 Is Required for the Maintenance of the Spindle Assembly Checkpoint and Chromosomal Stability. Int. J. Biochem. Cell Biol..

[35] Mishra R.K., Chakraborty P., Arnaoutov A., Fontoura B.M.A., Dasso M. (2010). The Nup107-160 Complex and Gamma-TuRC Regulate Microtubule Polymerization at Kinetochores. Nat. Cell Biol..

[36] Del Viso F., Huang F., Myers J., Chalfant M., Zhang Y., Reza N., Bewersdorf J., Lusk C.P., Khokha M.K. (2016). Congenital Heart Disease Genetics Uncovers Context-Dependent Organization and Function of Nucleoporins at Cilia. Dev. Cell.

[37] Jao L.-E., Akef A., Wente S.R. (2017). A Role for Gle1, a Regulator of DEAD-Box RNA Helicases, at Centrosomes and Basal Bodies. Mol. Biol. Cell.

[38] Busayavalasa K., Chen X., Farrants A.-K.Ö., Wagner N., Sabri N. (2012). The Nup155-Mediated Organisation of Inner Nuclear Membrane Proteins Is Independent of Nup155 Anchoring to the Metazoan Nuclear Pore Complex. J. Cell Sci..

[39] Hawryluk-Gara L.A., Shibuya E.K., Wozniak R.W. (2005). Vertebrate Nup53 Interacts with the Nuclear Lamina and Is Required for the Assembly of a Nup93-Containing Complex. Mol. Biol. Cell.

[40] Pitzen V., Sander S., Baumann O., Gräf R., Meyer I. (2021). Cep192, a Novel Missing Link between the Centrosomal Core and Corona in *Dictyostelium* Amoebae. Cells.

[41] Vollmer B., Schooley A., Sachdev R., Eisenhardt N., Schneider A.M., Sieverding C., Madlung J., Gerken U., Macek B., Antonin W. (2012). Dimerization and Direct Membrane Interaction of Nup53 Contribute to Nuclear Pore Complex Assembly. EMBO J..

