# Supplementary figures and images for: Partial Disassembly of the Nuclear Pore Complex Proteins during Semi-Closed Mitosis in Dictyostelium discoideum
Source: Cells. 2022 Jan 25;11(3):407. doi: 10.3390/cells11030407 (PMC8834467; doi:10.3390/cells11030407)

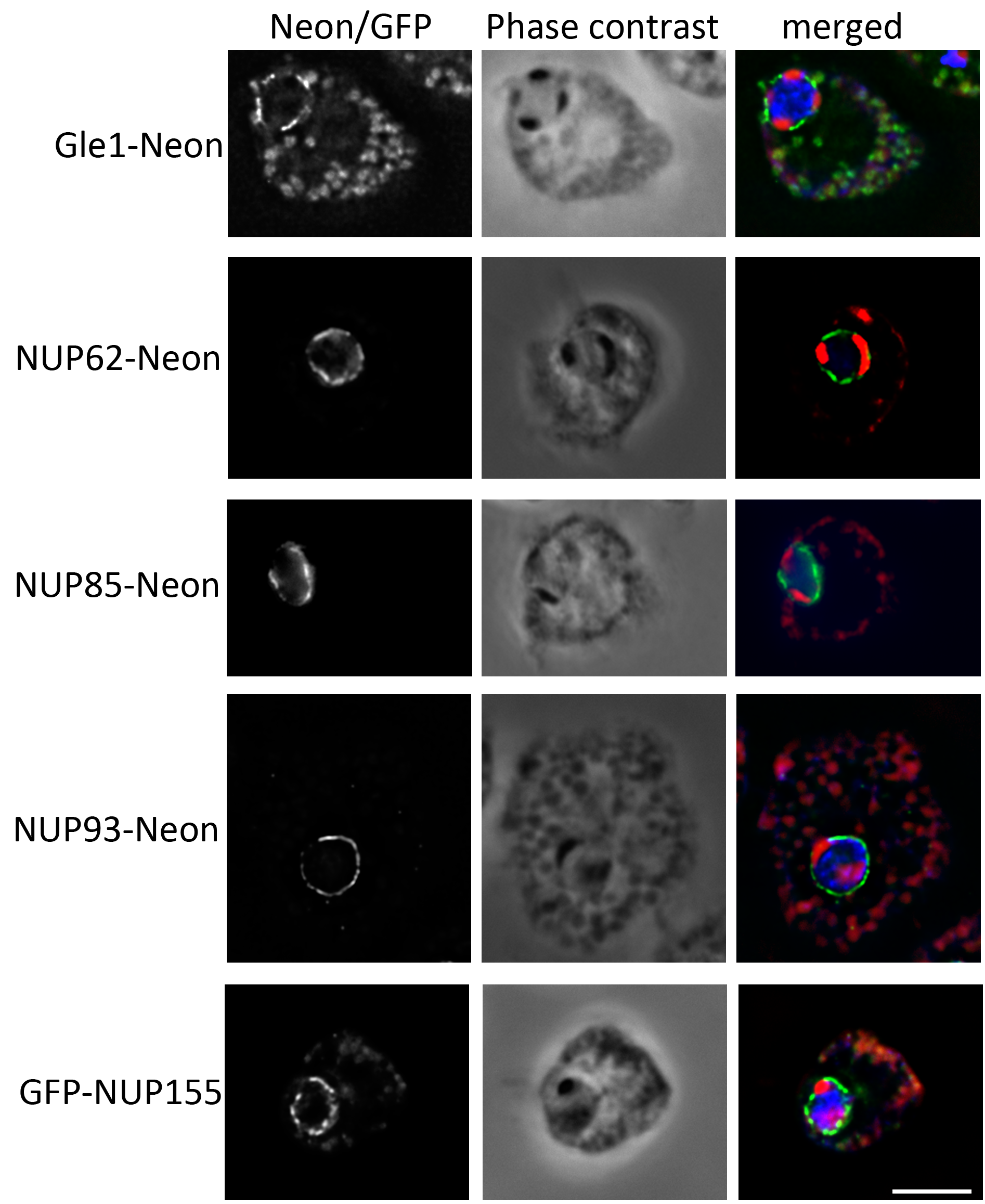

Supplement: Supplementary file 1 [file cells-11-00407-s001.zip › Figure S1.tif]

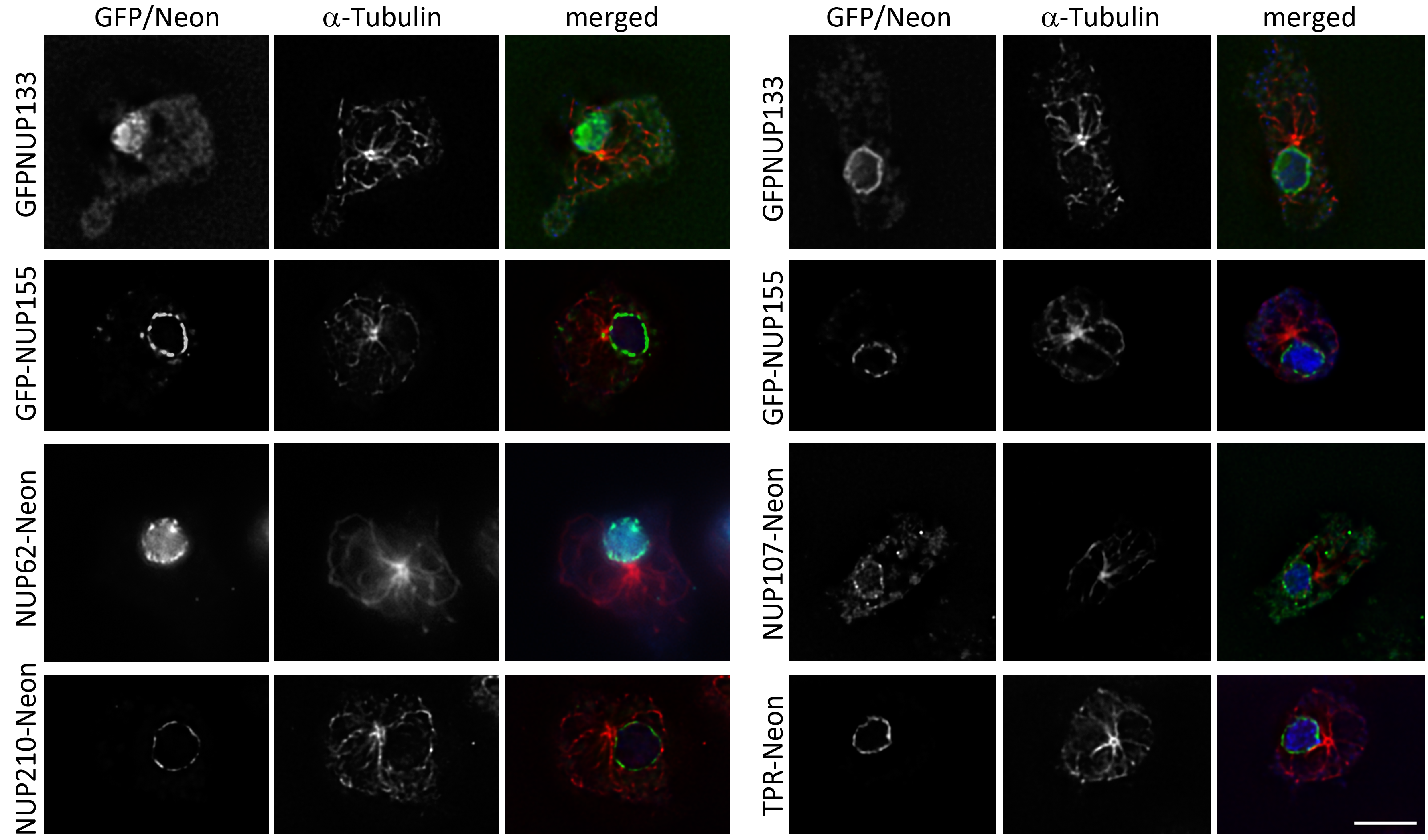

Supplement: Supplementary file 1 [file cells-11-00407-s001.zip › Figure S2.tif]

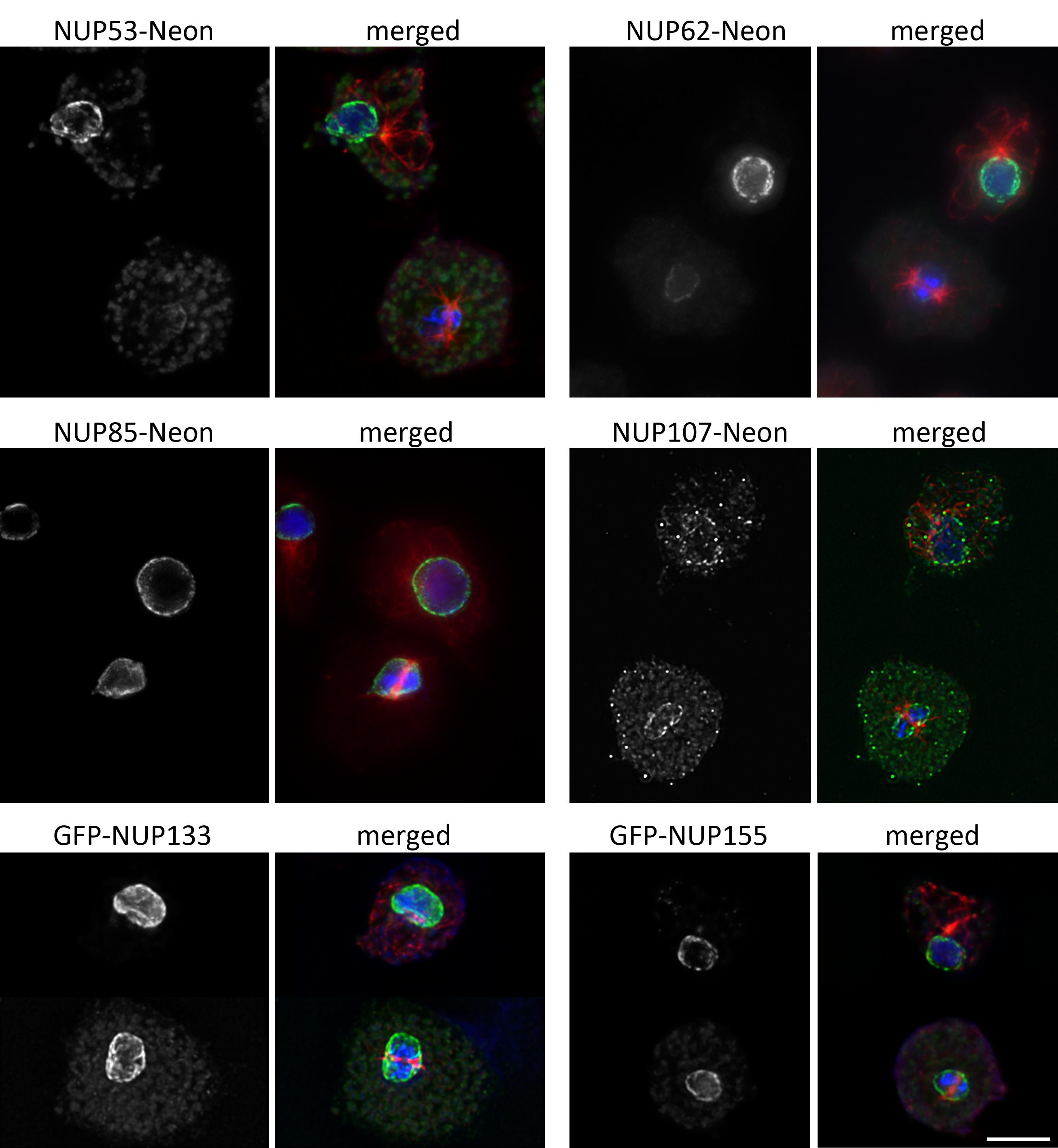

Supplement: Supplementary file 1 [file cells-11-00407-s001.zip › Figure S3.tif]

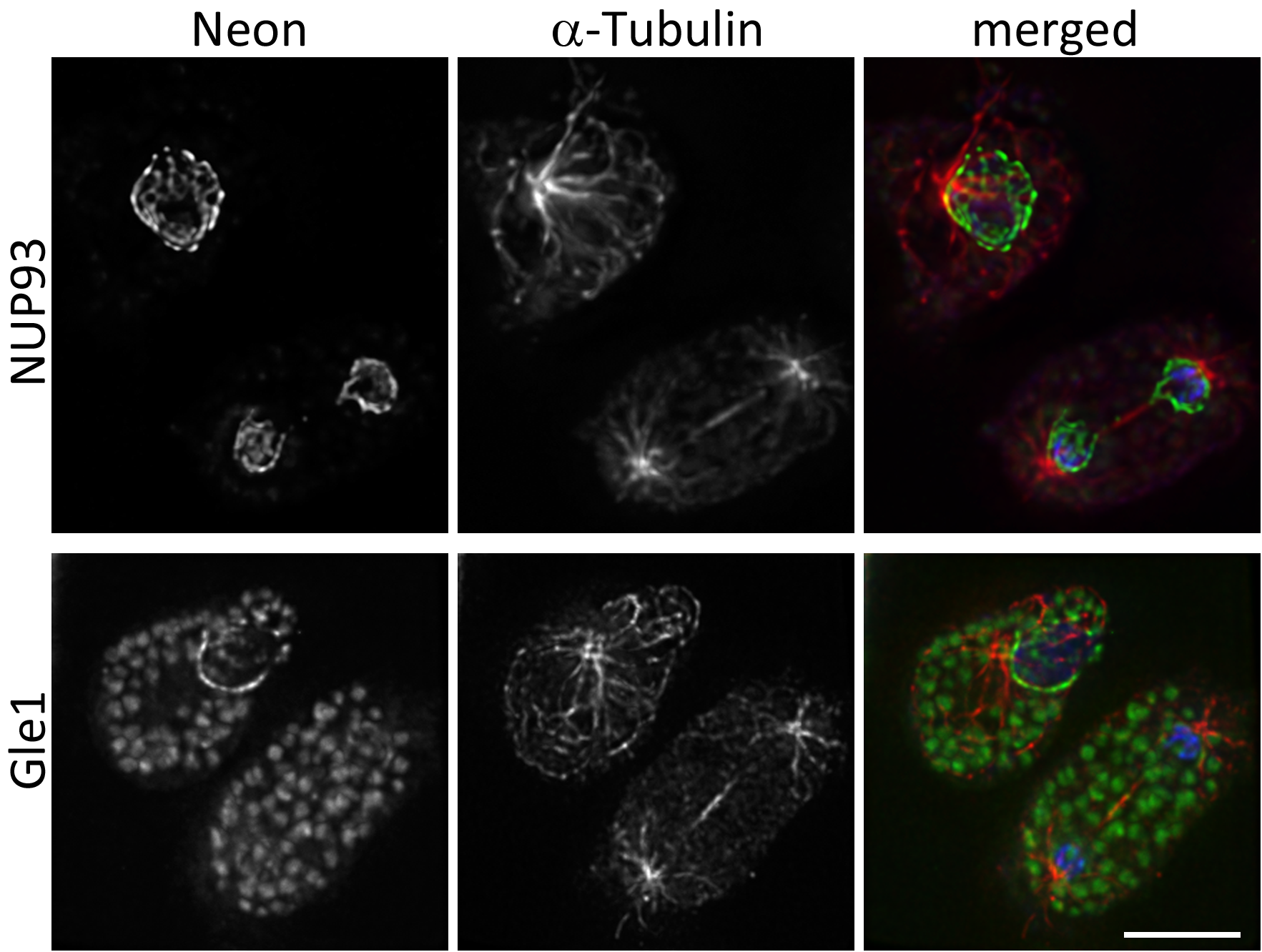

Supplement: Supplementary file 1 [file cells-11-00407-s001.zip › Figure S4.tif]
